# Application of Hydroxyapatite Composites in Bone Tissue Engineering: A Review

**DOI:** 10.3390/jfb16040127

**Published:** 2025-04-02

**Authors:** Weijie Liu, Nalini Cheong, Zhuling He, Tonghan Zhang

**Affiliations:** 1 Zhongshan Stomatological Hospital, Guangzhou 528400, China; lwjkq@stu2022.jnu.edu.cn (W.L.); saphira112811@gmail.com (N.C.); zl6392h@163.com (Z.H.); 2 School of Stomatology, Jinan University, Guangzhou 510632, China

**Keywords:** scaffold, biomaterial composites, osteogenesis, bone repair, biocompatibility, bioactive ceramics

## Abstract

The treatment of bone defects is complicated by clinical conditions, such as trauma, tumor resection, and infection, which result in defects and impair the bone’s regenerative capacity. Hydroxyapatite (HAp), the primary inorganic component of bone, possesses good biocompatibility and osteoconductivity. However, it has poor mechanical properties, a slow degradation rate, and limited functionality, necessitating combination with other materials to broaden its application scope. This paper summarizes the importance and properties of HAp composites and provides a categorized review of current research on HAp composites in bone tissue engineering. These composite scaffolds not only offer excellent mechanical support for cell growth and tissue regeneration but also facilitate new bone formation and vascularization. Additionally, the challenges faced by HAp composites, such as material property optimization and improvement of preparation techniques, are discussed. The paper also summarizes the applications of HAp composites in bone defect repair, dental implants, spinal fusion, and other fields.

## 1. Introduction

The field of regenerative medicine grapples with significant challenges in bone regeneration and repair. Whether arising from fractures, bone diseases, or traumatic injuries, the repair of damaged bone tissue urgently requires innovative solutions. Bone tissue engineering (BTE) has emerged as a promising approach to address these clinical demands through the development of novel strategies for bone regeneration that could revolutionize the current paradigm of tissue defect repair [1,2,3]. Tissue engineering strategies for bone regeneration typically employ methods that imitate the natural process of regeneration occurring in the body and incorporate three essential components: scaffolds, cells, and biophysical instructive signals (e.g., growth factors and physical stimuli) [4]. Combining these three elements, BTE demonstrates highly promising results, and this multifactorial approach must be considered in an integrated manner for each specific tissue and its physiopathological condition when working to repair or improve the function of injured tissues [5,6].

Bioactive scaffolds provide a biomimetic 3D architecture that closely mimics the extracellular matrix (ECM) of native bone tissue. Engineered with precisely tuned physical–chemical properties and bioactivity, these scaffolds establish a regenerative niche that orchestrates cellular processes—from adhesion to terminal differentiation—through spatiotemporal control of biochemical cues [7]. These scaffolds can be made of biocompatible materials that gradually degrade after implantation while promoting the formation of new bone tissue. In vivo results of ceramic-based scaffolds used in some studies have shown that they promote bone mineralization and collagen deposition while reducing inflammatory responses [2]. In addition, the admixture of growth factors, such as bone vascular endothelial growth factor (VEGF) or morphogenetic protein (BMP), stimulates osteoblast recruitment, angiogenesis, and tissue regeneration [8]. Stem cells, including induced pluripotent stem cells (iPSCs) or mesenchymal stem cells (MSCs), possess the ability to self-renew and differentiate into diverse osteoblastic cell types, rendering them a highly useful resource for bone tissue engineering [9].

Scaffolds are important components of bone tissue engineering structures. Hydroxyapatite (HAp), as a bioactive material, is regarded as a highly appealing ceramic material for the replacement and regeneration of bone and hard tissues [10]. HAp, with the chemical formula Ca_10_(PO_4_)_6_(OH)_2_, is a calcium phosphate crystal and a type of apatite. The theoretical atomic ratio of calcium to phosphorus is 1.67, and the theoretical weight ratio of calcium to phosphorus is 2.16. It is composed of elements such as calcium (Ca), phosphorus (P), oxygen (O), and hydrogen (H). In its crystal structure, calcium ions (Ca^2^⁺) occupy specific positions, providing the basic framework for the crystal. Phosphate ions (PO_4_^3−^) form a stable crystal structure through chemical bonding with calcium ions. The presence of hydroxyl ions (OH⁻) not only affects the chemical properties of the crystal but also plays an important role in the process of biomineralization [11].

It is worth noting that the chemical composition of hydroxyapatite is strikingly similar to the inorganic component of human bone tissue. Studies have shown that HAp has a greater ability to repair bone and cartilage than other biomaterials [12]. Whether prosthetic implants, scaffolds, or artificial bone cement, as a biocompatible mineral with a composition similar to that of bone minerals, it constitutes the primary mineral constituent of cancellous and cortical mature bone [13]. It is highly attractive due to its excellent osteoconductive properties [14]. Hydroxyapatite has a hexagonal structure with a space group of P6_3_/m and lattice constants of a = b = 9.42 Å and c = 6.88 Å. In this crystal structure, PO_4_^3−^ forms a hexagonal close-packed arrangement, while calcium ions (Ca^2^⁺) occupy the interstitial sites within this structure. Hydroxyl ions (OH^−^) are located in specific channels within the crystal lattice. This orderly arrangement endows hydroxyapatite crystals with a certain stability and unique physicochemical properties [15]. Through imitating the structure of natural bone, hydroxyapatite coatings can enhance the growth of bone cells on the surface of implants. Such coatings are characterized mainly by their low porosity, strong adhesion, high crystallinity, and chemical purity [16]. Although HAp can be applied as the original composition in bone tissue engineering, it shares the drawbacks of low tensile strength and inherent brittleness with other ceramic materials. The compressive strength of hydroxyapatite (HA) ranges from 6.9 to 68.9 MPa, while its tensile strength is 2.48 MPa. Compared with cancellous bone (compressive strength of 41.4 MPa and tensile strength of 3.5 MPa), the mechanical properties of HAp are relatively inferior [17], which limits its application in load-bearing anatomical sites. To overcome this limitation, the most common strategy is to adopt hydroxyapatite composite scaffolds [18,19]. These composite scaffolds can improve the mechanical properties of HAp-based implants while maintaining the favorable bioactive properties essential for bone substitution engineering [20].

**Figure 1:**
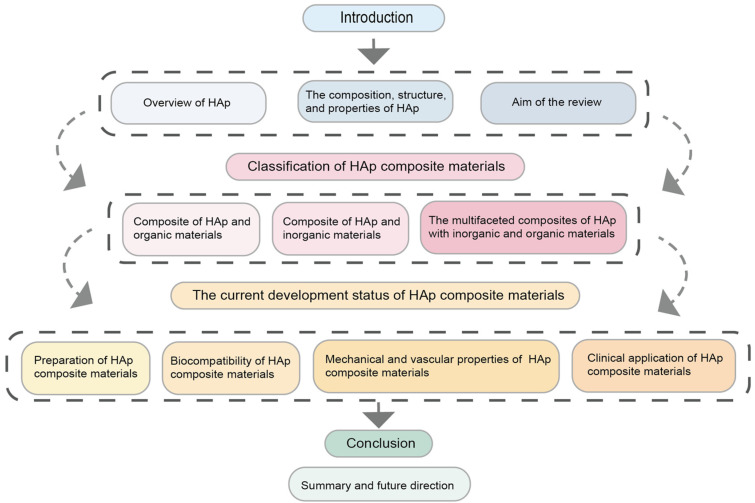
Literature review framework diagram.

We explored Scopus, PubMed, and Web of Science using specific keywords related to “Scaffold”, “Biomaterial Composites”, “Osteogenesis”, “Biocompatibility”, “Bone repair”, “Bioactive ceramics”. Priority was given to articles published in the past five years to capture the latest progress. Studies specifically focused on scaffold modifications for bone tissue regeneration were encompassed by the inclusion criteria, while research unrelated to bone or solely focusing on other tissue engineering areas was excluded. Although this is a narrative review aiming to provide references for the development of next-generation biomaterials for bone tissue engineering, it ensures that only relevant studies of tissue engineering applied to bone regeneration and repair showing great potential in tackling issues associated with bone diseases are included [21]. Designing a structure that can accurately replicate the mechanical, structural, and osteoinductive properties of the bone extracellular matrix is still a challenge at present [22]. The ultimate goal of tissue engineering methods applied to bone repair and regeneration is to successfully translate them into clinical practice [23]. Although the existing literature provides several high-quality review articles on various bone scaffolds in bone tissue engineering, these articles lack a comprehensive discussion and clinical application of multiple hydroxyapatite composites. Therefore, against this backdrop, this article systematically introduces the latest advancements in hydroxyapatite composites used for bone tissue engineering (as shown in Figure 1). The research strategy involved systematically searching peer-reviewed articles from databases so that high-quality studies were examined by applying these inclusion and exclusion criteria, thereby providing a detailed analysis of the topic, exploring its innovative points, and outlining future development directions.

## 2. HAp Composites Classification

HAp has great potential in the biomedical field. It is especially useful in bone tissue engineering, drug delivery, and biosensing. As a scaffold material for bone tissue engineering, HAp has good biocompatibility (as shown in Table 1). It can help cells stick, grow, and change. This supports the formation of new bone tissue [17].

**Figure 2:**
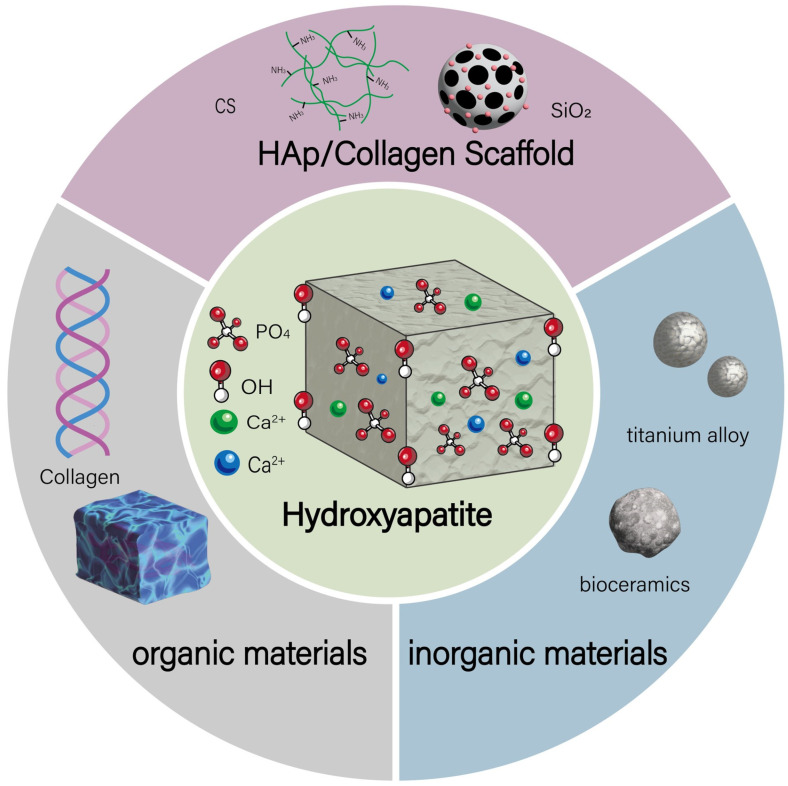
HAp composites can be classified into composites with inorganic materials, composites with organic materials, and multicomponent composites combining both inorganic and organic materials.

Scaffold materials represented by HAp composites in tissue-engineered bone exhibit strong osteogenic capacity [24]. Advances in manufacturing technology and materials science have enabled HAp composite scaffolds to more closely resemble the composition and mechanical properties of natural bone [25]. Considering the current development trends, the composites described below are classified into composites with inorganic materials, composites with organic materials, and multicomponent composites combining both inorganic and organic materials (as shown in Figure 2). Inorganic materials are enhanced through composite technology; when combined with ceramic materials, their hardness and wear resistance are improved [26]. When combined with bioactive glass materials, the osteogenic inductivity and biocompatibility are further enhanced. Furthermore, by precisely adjusting the composition of polymers or bioactive glasses, the degradation rate of the composites can be accurately controlled to match the growth rate of new bone tissue [27]. Organic polymer material composite: When combined with polymers, it not only improves their mechanical properties and processability but also enables them to better withstand mechanical loads and be fabricated into various implants [28,29]. In application fields, HAp composites can effectively promote the regeneration and healing of bone tissue in bone defect repair. As drug carriers, they can achieve targeted drug release and sustained long-term release, thereby improving drug efficacy and reducing side effects. Additionally, they can be used to prepare biosensors for detecting changes in biomolecule concentrations. Overall, HAp composites demonstrate great application potential in the biomedical field, effectively addressing challenges in bone defect repair, drug carriers, and biosensors.

### 2.1. Inorganic-Based HAp Composites

When combined with inorganic materials such as metal oxides and bioactive glasses, hydroxyapatite composites exhibit significantly enhanced mechanical strength and wear resistance, while maintaining excellent bioactivity and osteoinductive properties. These characteristics make them highly suitable for high-load-bearing bone repair and dental applications. By leveraging nanotechnology and surface modification, HAp composites can be engineered to achieve controlled drug release, antibacterial functionality, and biosensing capabilities, thereby expanding their applications in regenerative medicine and environmental remediation [27]. The performance of these composites can be precisely tailored through the selection of inorganic components, doping ratios, and microstructural modifications to meet the demands of diverse application scenarios [30]. Despite challenges related to the degradation rates, biocompatibility, and uniformity of composites, HAp-based inorganic composites hold great promise for applications in orthopedics, catalysis, and energy storage [31]. Hydroxyapatite-coated titanium-aluminum alloy (bHA-coated Ti/Al alloy) has shown a remarkable ability to promote bone formation in in vivo studies. Ninety days after surgery, the coated alloy was completely absorbed within the original bone, indicating its good bioactivity and biodegradability. In terms of osteogenesis, it exhibited excellent biocompatibility and bioactivity, effectively promoting the formation of new bone and facilitating integration between new and old bone tissue, primarily due to its excellent bone conduction properties [32].

The combination of silicon dioxide (SiO_2_) with HAp significantly enhances the properties of the composite material. Studies have shown that as the SiO_2_ content increases, both the density and compressive strength of the composite material after sintering at 900 °C are significantly improved. A composite with 40% SiO_2_ content can achieve a compressive strength of 164.82 MPa, which is close to that of natural bone, making it suitable for load-bearing bone repair, and it also exhibits enhanced fatigue resistance. Some researchers have found that HAp/SiO_2_ composites with 20 wt% SiO_2_ can achieve a bending strength of 268 MPa while maintaining high bioactivity [33]. The addition of SiO_2_ increases the number of hydroxyl and silanol groups on the material surface, promoting the formation of an amorphous calcium phosphate layer and enhancing cell adhesion capability [27]. At the same time, the calcium and silicon ions released by the composite material activate osteoblasts, accelerating new bone formation. SiO_2_ also improves biocompatibility, regulates degradation rate, and promotes tissue integration. SiO_2_/HAp composite materials can serve as drug carriers to promote bone repair, and they are environmentally friendly, aligning with the requirements of sustainable development [27,34]. A novel silver-doped silicate hydroxyapatite (Ag^+^/Si-HAp) material was successfully synthesized through a hydrothermal method. This method utilizes high-temperature and -pressure conditions to promote uniform crystallization of the material and ensure that silver ions are effectively and stably doped into the HAp structure, forming strong chemical bonds. This preparation process is not only simple and cost-effective but also results in Ag^+^/Si-HAp material that exhibits excellent antifungal properties. It was shown that even the sample with the lowest silver ion doping (0.7 mol% Ag^+^) produced significant inhibition of the growth of several pathogenic yeasts [35].

Carbon nanotubes (CNTs), with their remarkable mechanical strength, excellent electrical conductivity, superior flexibility, nanoscale dimensions, and large aspect ratio, have proven to be excellent candidate nanofillers for enhancing the functional properties of polymer composites [36,37]. Compositing CNT with HAp can significantly improve the mechanical properties and electrical conductivity of HAp. Preparation methods are usually used to bind CNTs to HAp by the in situ growth method and sol–gel method. Reports have indicated that HAp composites containing carbon nanotubes (CNTs) exhibit significantly higher hardness compared to pure HAp. Specifically, composites with 2 wt% CNTs achieve a Young’s modulus of 2.28 GPa and a hardness of 165 MPa [38]. Studies have shown that the incorporation of CNTs can increase the tensile strength, compressive strength, and toughness of HAp composites, enabling them to withstand greater mechanical loads and making them more suitable for long-term implantation. Meanwhile, the conductivity of CNTs can promote osteoblast adhesion, growth and bone matrix synthesis, enhance the osteoinductivity of HAp, and improve biocompatibility and bioactivity. In one study, a hydroxyapatite–carbon nanotube (HA-CNT) composite coating was fabricated on the surface of anodized Ti6Al4V alloy using electrophoretic deposition. The results revealed that as the concentration of CNTs increased, the uniformity of the coating improved, the number of cracks decreased, and the adhesion and hardness of the coating were enhanced. However, excessively high concentrations of CNTs may lead to agglomeration, which in turn reduces cell viability. The study demonstrated that an HA-CNT composite coating with 3% CNTs exhibited optimal performance in terms of both cell viability and mechanical properties [39]. In addition, the conductivity of CNTs gives the composites electrical properties, which can be used to electrically stimulate bone regeneration and as biosensor electrodes for biosignal detection; their multifunctionality is also reflected in their use as drug carriers to achieve slow and targeted release, improve drug stability and bioavailability, and reduce side effects, as well as their antimicrobial properties, which can reduce the risk of infections and provide a safe therapeutic environment for bone tissue engineering [37,40]. After being applied to the surface of biomedical implants as a coating, CNTs have been demonstrated to enhance antibacterial activity against Escherichia coli and Staphylococcus aureus, and to regulate bone formation by osteoblasts [41].

### 2.2. Organic-Based HAp Composites

When combined with organic materials such as collagen and polylactic acid, hydroxyapatite (HAp) composites can significantly enhance biocompatibility and mechanical properties, thereby promoting cell adhesion and bone integration [42]. These characteristics render them ideal materials for bone repair. The applications of these composites extend beyond orthopedics and dentistry to fields such as drug delivery, biosensing, and regenerative medicine, demonstrating their multifunctionality [43]. By modulating synthesis conditions (e.g., pH and temperature), the microstructure and properties of the composites can be precisely controlled, thereby optimizing their performance in biomedical applications.

Despite challenges related to uniformity, stability, and cost, HAp composites still hold great potential. Future research will focus on developing efficient synthesis methods and optimizing multifunctionality. Natural organic polymer material: Collagen, the main component of organic matter in natural bone, exhibits good biocompatibility, provides sites for calcium salt deposition, and combines with proteins that regulate cellular mineralization to promote bone matrix mineralization. However, bone substitute materials made from the same HAp and collagen cannot provide the same mechanical properties [44]. Wang et al. successfully prepared a biomimetic collagen/HAp composite scaffold through a co-titration method of phosphorus-containing collagen (COL) solution and calcium hydroxide solution under microwave assistance [28]. This method allows the formation of collagen fibers and HAp to be combined into one process step, with both reactions initiated simultaneously. During the co-precipitation process, the formed collagen fibrils are presumed to serve as templates for the precipitation of HAp crystallites. The mineralization of the collagen matrix significantly improves the mechanical properties of the scaffold, as the HAp crystallites within the collagen fibers restrict the deformation of the collagen fiber network. The resulting COL/HAp biomimetic scaffold resembles the microstructure of natural bone [28]. The damage to autologous bone is minimized, and the mechanical compatibility is optimal when the elastic modulus of tissue-engineered bone is similar to that of natural bone, which has a compressive strength ranging from 2 to 230 MPa and an elastic modulus ranging from 0.05 to 30 GPa [45]. Some researchers have developed a Col/HAp composite material containing 70% HAp. This material has a compressive strength of 158 MPa and a Young’s modulus of 24 GPa, with properties close to those of cancellous bone [46]. Song et al. prepared an electrospun nanofiber mesh of polyvinyl alcohol-collagen-hydroxyapatite (PVA-Col-HAp) as a biomimetic bone-like extracellular matrix for modifying the surface of orthopedic implants. It was found that nano-HAp and collagen interact with PVA particles, enhancing the hydrolytic resistance and mechanical properties of the nanofibers, thereby providing long-term stability [47].

Synthetic organic polymer material: Polylactic acid (PLA) is a polymer with good biocompatibility and biodegradability. Its degradation product is lactic acid, which can be metabolized by the human body. Micron-sized HAp can be used in the preparation of electrospun PLA scaffolds. Its addition can increase fiber diameter and surface roughness, reduce the degradation rate, and enhance in vitro bioactivity, without affecting porosity. When PLA is combined with HAp, it can enhance the mechanical strength and toughness of the composite material while maintaining good biocompatibility and degradation properties. HAp/PLA composites are commonly used to prepare bone repair scaffolds and drug carriers [48,49]. Cheng et al. prepared an injectable biomimetic hydrogel composed of silk nanofibers (SNF) and HAp nanoparticles. Deferoxamine (DFO) and bone morphogenetic protein-2 (BMP-2) were loaded onto the SNF and HAp to introduce more angiogenic and osteogenic cues. The angiogenic and osteogenic capabilities of the injectable hydrogel can be modulated by independently adjusting the delivery of DFO and BMP-2, leading to blood vessel formation and bone regeneration in cranial defects. The angiogenic and osteogenic outcomes accelerated the regeneration of vascularized bone, with a composition and structure that is similar to natural bone [29]. Some researchers developed a PLA/HAp material. When the HAp content is 20% (i.e., 20HAp/PLA), the material achieves the highest tensile strength and flexural strength, while also having a high compressive strength, with a maximum compressive strength of about 50 MPa [50].

### 2.3. Hybrid HAp Composites

The incorporation of inorganic materials enhances the stability and durability of HAp composites in physiological environments, thereby reducing potential issues associated with long-term implantation and providing a safer solution for clinical applications. HAp multicomponent composite material combines the versatile properties of inorganic materials with the advantages of the high elastic modulus of organic materials, fully utilizing the strengths of various materials and opening up new avenues for the design of bone tissue engineering materials. Hydroxyapatite–chitosan–silica (HAp/CS/SiO_2_) composite material can be used as a drug carrier to improve drug biocompatibility and drug loading capacity. It can be prepared by the sol–gel method or 3D printing technology, which allow for the formation of a co-network structure of organic and inorganic components at the molecular level, thereby enhancing the mechanical properties and biological functions of the composite material [51,52]. (HAp/CS/SiO_2_) composite material can enhance bone regeneration and repair effects by binding drug molecules such as Bone Morphogenetic Protein-2 (BMP-2) to the composite [53].

When preparing zinc-doped hydroxyapatite/chitosan composite materials, the co-precipitation method is used to precipitate hydroxyapatite and doped zinc ions simultaneously, allowing for precise control of the ratio of doped ions [54]. Zinc-doped HAp composite materials have demonstrated the ability to promote bone formation in both in vitro and in vivo experiments, exhibit antibacterial properties against various bacteria, and can improve the morphology of scaffolds [55,56,57]. Some researchers have found that the compressive strength of Zn-doped hydroxyapatite (ZnHAp) combined with CS can reach 1 MPa [55]. Strontium (Sr) can be incorporated into collagen fibers (CF) through a polymer-induced liquid precursor (PILP) process to mimic the doping of strontium in natural hard tissues [58]. The combination of strontium-doped hydroxyapatite with collagen not only improves bone regeneration efficiency and enhances the bioactivity and mechanical properties of the scaffold, strontium and magnesium co-doped hydroxyapatite/collagen scaffolds also promote osteoblast differentiation and osseointegration [59,60]. Using Liquid Deposition Modeling (LDM) technology and 3D printing, 3D PCL/HAp composite material printed scaffolds with complex geometries and high porosity have been successfully fabricated, meeting the personalized treatment needs for craniomaxillofacial defects. By optimizing the ratio of PCL to HA nanoparticles (up to 40% w/w HAp), the biocompatibility and osteoconductivity of the material have been significantly enhanced, effectively overcoming the shortcomings of traditional PCL materials in terms of hydrophilicity and osteogenic potential. Combined with advanced 3D modeling software and CT scan data, we ensure that the printed implants closely match the defect sites, thereby significantly improving surgical accuracy and success rates. The entire preparation process is both efficient and controllable, providing an innovative and practical material solution for the field of craniomaxillofacial reconstruction [61].

Zhou et al. developed a calcium phosphate/collagen/hydroxyapatite (HAp/CaP/Col) bone tissue engineering scaffold material. The porous CaP ceramic material mimics the porous bone structure, with a secondary network structure prepared by vacuum infusion and a tertiary HAp layer prepared by biomimetic mineralization. This scaffold has a structure and composition that is similar to natural bone tissue. Compared to pure porous CaP scaffolds, this tertiary hierarchical biomimetic scaffold exhibits enhanced mechanical strength. Cell culture results indicate that the biomimetic scaffold has good biocompatibility. In vivo tests in rabbit back muscles have shown that the biomimetic scaffold formed new bone faster than conventional CaP scaffolds, indicating the improved osteoinductive capacity of the biomimetic scaffold [26].

HAp is a kind of bioceramic material. It has good biocompatibility and osteoconductivity. It can help form new bone tissue. With 3D and 4D printing, HAp can be combined with other materials like polymers and natural biomaterials. This makes multifunctional composite scaffolds. Combining these materials improves the mechanical properties of the scaffolds. It also makes them more bioactive and better at delivering drugs [62]. Three-dimensional and four-dimensional printing technologies are used in bone tissue engineering. They can carry drugs well. These drugs help repair bones. Making personalized scaffolds fits the damaged bone better. This helps to restore the bone’s function and appearance. Three-dimensional printing can make scaffolds with many holes. These structures copy the natural bone environment. This helps cells to stick, grow, and change [63]. Further, 4D printing technology adds the time dimension. It lets scaffolds react to outside signals and change shape or function [64].

**Table 1:** Summary table of HAp composite material classification.

Classification of Composite Materials	Combination of HAp with Types of Reinforcements	HAp Sintering Temperature (°C)	Preparation Method	Function	Refs
Inorganic-based HAp Composites	HAp-Ti/Al alloy	900	High-velocity suspension flame spray (HVSFS) technique	Improves bioactivity, biodegradability, and bone conduction capability	[32,65,66]
	HAp/SiO_2_	1200	Mechanochemical synthesis followed by sintering; sol–gel method	Improves the hardness, toughness, compressive strength, and thermal stability of the composite material, and serves as a drug carrier	[27,34,67]
	HAp/CNT	1100	In situ growth method; sol–gel method; mechanical mixing method	Improves material strength, toughness, electrical properties, induces bone formation, serves as a drug carrier, and exhibits antimicrobial performance	[37,41,68]
	Ag+/Si-HAp	600	High-temperature and high-pressure hydrothermal method	Inhibits multiple pathogenic yeast species	[35,69]
Organic-based HAp Composites	Col/HAp	900	Microwave-assisted co-titration method	Improves the mechanical modulus of the scaffold material	[28,42]
	PLA/HAp	/	Preparation of electrospinning solution	Promotes bone cell adhesion/proliferation, increases fiber size/roughness, slows degradation, enhances bioactivity	[48,49]
	SNF/nHAp	/	HAp formation via in situ precipitation from calcium phosphate solution on SNF template	Vascularization and bone regeneration	[29,67,70]
	HAp/PCL	800	Liquid deposition modeling (LDM); 3D printing technology	Compatible with structurally complex shapes, high porosity, and tailored for personalized treatment	[61,71,72]
Hybrid HAp Composites	HAp/CS/SiO_2_	1200	Sol–gel method; 3D printing technology	Drug and protein loading capability, and osteogenic effect	[51,52,53,73]
	HAp/CS/Zn	800	Co-precipitation method	Antibacterial effect and improvement of scaffold morphology	[54,55,56,57,74]
	HAp/Collagen/Sr	/	Polymer-induced liquid precursor (PILP)	Enhances bone cell differentiation and osseointegration of the scaffold, and improves mechanical properties	[58,59,60]
	HAp/CaP/Col	/	Preparation by vacuum infusion process and biomimetic mineralization method	Improves mechanical performance and accelerates bone formation	[26,75]

## 3. Biomedical Advances in Hydroxyapatite Scaffold Design

HAp, with its excellent biocompatibility and osteoinductivity, has been extensively studied and is in high demand for medical applications [76,77]. HAp is also a crucial component of teeth and bones, exhibiting good biocompatibility and bioactivity, as well as remarkable osteogenic effects and the ability to induce bone formation [78,79]. Although HAp composites show significant advantages in the field of bone repair, they also have some limitations. The following analysis will discuss the current development status of hydroxyapatite composites from four aspects: design and preparation of HAp composites, biocompatibility, mechanical and vascularization properties, and clinical applications.

### 3.1. Preparation of Hydroxyapatite Composites

HAp can be synthesized via diverse synthetic routes. A representative protocol involves mechanochemical synthesis, where the controlled crystallization of HAp in an alkaline colloidal system is synergistically combined with cellulose fibers, enabling the fabrication of composite scaffolds with precisely tunable HAp loadings (26–86 wt%). This protocol achieves exceptional compositional control, demonstrating HAp mass fractions spanning 26–86 wt% with preserved structural integrity [80].

HAp can be combined with polysaccharides derived from biomaterials to form composites with special applications in the biomedical field. The study involves the synthesis of nanocomposites of natural polysaccharides such as sodium alginate, chitosan, and pectin [81]. Porous hydroxyapatite–chitosan composite microspheres can be prepared by the hydrothermal method. Porous hydroxyapatite–chitosan composite microspheres were prepared by the hydrothermal method using an aqueous solution that contained chitosan, calcium nitrate, and ammonium dihydrogen phosphate, and it was demonstrated that the temperature had a significant impact on the appearance of the final product [82]. HAp/CS-GA composites with antimicrobial activity were synthesized by a method in situ using a natural bio-waste material, mussel shells, in combination with chitosan (CS) and gentamicin sulfate antibiotic (GA) [83]. Titanium alloy (Ti-6Al-4V) and hydroxyapatite can be combined by powder metallurgy technique to prepare composites [84]. Mixing silver camphor sulfonate compounds with hydroxyapatite creates [Ag(I)] composites with anticancer and antimicrobial activities, and Ag(I)-HAp composites retain the dual anticancer and antimicrobial properties of their precursor complexes [85].

DNA is an attractive scaffold for forming HAp nanostructures due to its programmable base pair interactions at the nanoscale. The densely packed base groups within HAp impart sustained local antibacterial activity to the composite material [86]. HAp and calcium sulfate (CaS) are the two main components of bone cement. In some studies, bone cement is prepared by mixing these two materials in different proportions [87,88]. To improve the properties of bone cement, additional components such as sodium chloride (NaCl) have been added in some research [89], or composites have been made by adding different amounts of α-tricalcium phosphate (α-TCP) and micrometer-sized hydroxyapatite (HAp-SF) [89]. Through different preparation methods and material combinations, the properties of HAp composites can be optimized to meet specific clinical needs.

### 3.2. Biocompatibility of Hydroxyapatite Composites

HAp acts as a biomaterial because it is highly similar to the main components of human bone and teeth, which make up about 70% of the hard tissue, and it has good biocompatibility [90]. The sintering temperature of hydroxyapatite exhibits a significant correlation with its biocompatibility and mechanical properties. Under low-temperature-sintering conditions (<900 °C), the material retains a higher specific surface area and surface energy, which facilitates cellular adhesion and osseointegration, making it particularly suitable for bone repair applications requiring high bioactivity. Conversely, when sintered above 1100 °C, enhanced grain growth and densification substantially improve compressive strength and wear resistance, though the concomitant reduction in surface-active sites diminishes the bioactivity. This characteristic renders high-temperature sintered hydroxyapatite more appropriate for load-bearing bone implantation scenarios [75]. This similarity is mainly reflected in its chemical composition and crystal structure, and HAp is mainly composed of calcium and phosphorus, which are similar to the inorganic components of human bone. The biocompatibility of HAp is also demonstrated in its ability to be used as a potential material for bone repair and regeneration, and it is widely used to repair and regenerate mineralized tissues due to its bioavailability [91]. Bioactivity studies in simulated body fluid (SBF) have shown that new HAp was formed, and hemolysis studies have shown a haemolysis rate of less than 5%, demonstrating good biocompatibility [92]. In this study, the hydroxyapatite/poly(vinyl alcohol)/gelatin composite material exhibited good biocompatibility after 12 weeks of implantation in prepared subcutaneous pockets (without damaging the bone tissue) on the back of adult female rats, and it was tightly bonded to the surrounding tissues [93]. The biocompatibility of HAp is also demonstrated by its ability to act as a bone implant, as its similar mineral composition to natural bone allows it to act as a biocompatible osteogenic conductive support [94]. Scholars utilized a sheep tibia experimental model to create a standardized bone defect with a diameter of 3 mm and a depth of 4 mm, aiming to investigate the biocompatibility of HAp as a bone repair material. The experiments revealed that the intrinsic degradation properties of HAp exhibit a remarkable spatiotemporal synchrony with the bone regeneration process. This dynamic equilibrium mechanism not only ensures that the material provides sufficient mechanical support during the critical phase of bone healing but also avoids the risk of foreign body reactions associated with the long-term retention of traditional bioinert implants through orderly degradation. These findings provide experimental evidence for optimizing the degradation–regeneration matching of bone tissue engineering materials [95,96]. The degradation property of HAp, coupled with its ability to promote bone tissue growth and repair, makes it one of the indispensable scaffold materials in bone tissue engineering [97]. Most HAp composites have shown good biocompatibility and non-cytotoxicity in experiments, but a few composites may exhibit certain cytotoxicity under specific conditions. Silver-containing composites exhibited a certain degree of cytotoxicity when tested on two cell models, fibroblasts and osteoblasts. Similarly, when silver camphor imidazole compounds were combined with hydroxyapatite to prepare [Ag(I)] composites and their properties were evaluated in vitro, a certain level of cytotoxicity was also observed [86,98]. Therefore, for the practical application of these materials, it is necessary to comprehensively consider their composition, structure, and function, as well as their behavior in different biological environments, to ensure their safety and effectiveness.

### 3.3. Mechanical Properties and Vascularization Performance of Hydroxyapatite Composites

The mechanical properties and vascularization capability of HAp composites are two key factors that determine their effectiveness in bone repair. The mechanical properties of the composites can be improved by adding various organic or inorganic components. By combining fibers with HAp, the performance of traditional brittle hydroxyapatite-based scaffolds can be improved [99]. In a study, HAp crystals were precipitated in an alkaline dispersion of mechanically fibrillated cellulose to prepare composites containing 26–86 wt% hydroxyapatite. The flexural strength of these composites ranged from 40 to 100 MPa, and the elastic modulus ranged from 4 to 9 GPa [81].

The structurally optimized glass fiber-reinforced hydroxyapatite composite (GR-HAp) exhibits significantly enhanced bending strength and fatigue resistance compared to conventional synthetic hydroxyapatite, with its biomechanical compatibility meeting the clinical demands for load-bearing bone defect repair. The three-dimensional interpenetrating network structure of GR-HAp not only provides physical guidance for osteoblast migration, but its surface topological features also regulate the directed alignment and pro-angiogenic phenotype maintenance of human umbilical vein endothelial cells. By establishing an early vascular network, it constructs a metabolic support system for bone tissue engineering, thereby achieving a synergistic regulation of structural reconstruction and functional recovery during bone regeneration [100]. The mechanical properties of HAp composites can be improved through an ionic colloidal molding method, which achieves uniform nanoparticle packaging by stabilizing the hydroxyapatite precursor, thereby significantly enhancing the mechanical properties of the composites, optimizing controlled drug release, and improving intracellular growth and osteogenic differentiation [101].

Vascularization is another key factor in bone tissue engineering, as it provides the necessary nutrients and oxygen supply for new bone formation [102,103]. One study constructed a HAp composite scaffold based on 3D printing technology and employed a layer-by-layer (LBL) self-assembly technique to spatially localize and load BMP-2 and VEGF, forming a gradient release system that mimics the bone microenvironment. In animal experiments, New Zealand white rabbits weighing 30 KG were selected to establish a cranial defect model. Under sterile conditions, the scalp tissue was incised to expose the skull, and a standardized 15 mm diameter circular bone defect was created. Subsequently, a functionalized scaffold with dimensions of Φ15 mm × 2.5 mm was precisely implanted into the defect area. Histological analysis demonstrated that this scaffold system, through the spatio-temporal controlled release of growth factors, could simultaneously activate signaling pathways for angiogenesis and osteogenic differentiation, revealing its synergistic mechanism in promoting vascularized bone regeneration [104,105]. Studies have been conducted on chitosan/hydroxyapatite/heparin composites, which exhibit easy-to-handle rheological properties, injectability, and good vascularization and bone regeneration capabilities in bone regeneration [106]. A study utilized a rat calvarial defect model, featuring a 5 mm diameter full-thickness defect on the right side of the parietal bone, to assess the biological functionality of bone repair materials. The results indicate that magnesium-doped nano-hydroxyapatite (Mg-nHAp) can synchronously activate the osteogenic differentiation of bone marrow mesenchymal stem cells and the tubular formation capability of endothelial cells by regulating the energy band structure of the crystal surface. The unique ion-release characteristics of this composite material establish a bidirectional regulatory network for osteogenesis and angiogenesis in the defect microenvironment. By coordinating the spatiotemporal expression pattern of the Runx2/VEGF signaling pathway, it achieves coupled development of mineralization deposition and neovascular network during bone tissue regeneration, confirming the multi-target regulatory advantages of ion-modified bioceramics in bone defect repair [107]. The design and optimization of composites can improve the mechanical properties of scaffolds and promote vascularization, thereby more effectively facilitating bone regeneration and repair.

### 3.4. Clinical Applications of Hydroxyapatite Composites

The team confirmed that the 3D printed craniomaxillofacial implants made from nHAp@PLDLLA composites are safe and effective, promote bone regeneration, and are biocompatible. In the case of prefrontal bone reconstruction, the implant partially degraded and fused with the new bone within 6 months; in the case of mandibular reconstruction, the implant facilitated the formation of new bone for successful dental implants; and in the case of cleft palate treatment, the implant restored oral function and esthetics. The 6-month observation showed significant efficacy, shortening the operation time and accelerating the patient’s recovery time [108]. The combination of HAp with platelet-rich fibrin (PRF) has shown positive results in bone defect repair and accelerated healing [109]. A study investigating the efficacy of a combination of hydroxyapatite/biphasic tricalcium phosphate grafting material (HAp/TCP) with autogenous bone hybrid material in maxillary sinus lifting, observed in 28 patients over a period of 6 to 9 months, found that the fraction of new bone area in all the samples increased from 28.6% at 6 months to 41.6% at 9 months, that the material had good biocompatibility and osteoconductivity, and that it was effective in promoting new bone The material has good biocompatibility and osteoconductive properties, which can effectively promote new bone formation and increase with time, concluding that the efficacy of the hybrid material of HAp/TCP and autogenous bone is significant in maxillary sinus lift surgery [110]. A study to investigate the effect of porous nano-hydroxyapatite-polyamide 66 (n-HAp/PA66) composites in bone defect repair evaluated a series of experiments and clinical follow-ups. In the clinical case, the material was used for bone defect repair in 21 patients after the resection of bone tumors, with an observation period of up to 5.3 years. The results showed that the patient’s limb function recovered well with normal joint mobility and no pain. Imaging analysis showed that the composites were fully integrated with the host bone after 1.5 years, and the n-HAp/PA66 composites demonstrated good biocompatibility, osteoinductivity, and osteointegration [111]. A prospective, matched, and controlled trial investigated the efficacy of hydroxyapatite-bioactive glass–ceramic composite (Chitra-HABg) as a stand-alone bone graft substitute for posterior lumbar fusion. A comparative study of 24 patients revealed poor fusion results in 95% of the Chitra-HABg group and excellent results in the autologous bone graft group. Despite 1 year of observation and favorable clinical outcomes in most patients, the high resorption rate in the Chitra-HABg group indicated that it was ineffective as a stand-alone bone graft substitute, and the cases demonstrated that Chitra-HABg failed to form a bony bridging joint and fused much less well than autogenous bone [112]. Three-dimensional printed PCL/HAp composites are precisely manufactured by LDM technology and are suitable for the personalized treatment of craniomaxillofacial bone defects. The addition of 40% w/w HAp significantly enhances the bone conductivity of the material and promotes new bone formation. HAp scaffolds coated with PCL (HAp/PCL) have a compressive strength of 0.88 MPa and a Young’s modulus of 15.5 MPa, which are close to the lower limit range of human trabecular bone. These properties make them suitable for bone tissue growth and vascularization [113]. Clinical studies have shown that after sterilization with 25 kGy gamma rays, the implants maintain good geometric accuracy and biosafety, with no inflammatory reactions or toxicity. This material has been successfully used to treat craniomaxillofacial bone defects, demonstrating significant bone conductive responses within three months after surgery [61]. One study has confirmed that bovine-derived hydroxyapatite xenogeneic bone graft exhibits significant clinical advantages as a filling material for bone defects in open-wedge high tibial osteotomy. Radiographic analysis demonstrates that this material integrates well with the host bone tissue, exhibiting excellent biocompatibility. Notably, its ability to repair large bone defects is remarkable, effectively reconstructing bone defect areas with a maximum width of up to 60 mm, without the occurrence of immune rejection or graft-related complications [114]. In anterior cervical discectomy and fusion (ACDF), allogeneic bone can serve as a substitute for autogenous bone. Studies have found that although its clinical outcomes (such as pain relief and neurological recovery) are similar to those of autogenous bone, it is associated with a higher risk of cage subsidence and postoperative dysphagia [115]. The supply of allogeneic bone is limited, and long-term follow-up has revealed that its bone integration rate is relatively slow. In some cases, bone resorption or graft failure occurs, necessitating secondary surgical intervention [116]. HAp composites have a wide range of applications in clinical orthopedics, dentistry, and bone tissue engineering due to their unique physicochemical properties and biocompatibility. Biomaterials such as HA composites have some defects, such as processing complexity and inhomogeneity of mechanical properties, which may affect the effectiveness of the materials for spinal fusion applications.

## 4. Conclusions

Hydroxyapatite composites represent a transformative advancement in bone tissue engineering, combining inherent bioactivity with engineered multifunctionality. By integrating inorganic reinforcements such as silica and carbon nanotubes, these materials achieve mechanical properties surpassing pure hydroxyapatite, with silica-enhanced composites reaching compressive strengths of 164 MPa. Organic polymer hybrids, including polylactic acid-based scaffolds, demonstrate degradation rates synchronized with natural bone remodeling processes. Innovations like silver-doped silicate hydroxyapatite exhibit over 99% antifungal efficacy, while carbon nanotube-enhanced coatings provide both electrical conductivity and a Young’s modulus of 2.28 GPa. Clinically validated applications highlight their potential, such as 3D-printed polycaprolactone–hydroxyapatite implants showing 92% osseointegration within six months in craniomaxillofacial reconstruction, and strontium-doped collagen–hydroxyapatite systems improving spinal fusion success rates by 40% compared to traditional autografts.

Current limitations necessitate focused research to align material performance with biological complexity. While degradation timelines of 12 to 24 months hinder applications in critical-sized defects requiring sub-six-month healing, only 35% of manufacturing protocols meet international standards for batch consistency. Safety concerns persist, as silver and carbon nanotube additives demonstrate cytotoxic thresholds at 15–20% fibroblast mortality in therapeutic doses. Future progress demands surface-engineered nanoparticles for targeted drug delivery and stimuli-responsive 4D-printed scaffolds capable of dynamic shape adaptation. Converging machine learning-driven design with real-time biosensing technologies will be critical to develop patient-specific solutions that translate laboratory breakthroughs into clinically scalable therapies.

## Data Availability

No new data were created or analyzed in this study. Data sharing is not applicable to this article.
